# Whole Genome Sequencing of Elite Rice Cultivars as a Comprehensive Information Resource for Marker Assisted Selection

**DOI:** 10.1371/journal.pone.0124617

**Published:** 2015-04-29

**Authors:** Jorge Duitama, Alexander Silva, Yamid Sanabria, Daniel Felipe Cruz, Constanza Quintero, Carolina Ballen, Mathias Lorieux, Brian Scheffler, Andrew Farmer, Edgar Torres, James Oard, Joe Tohme

**Affiliations:** 1 Agrobiodiversity research area, International Center for Tropical Agriculture, Cali, Colombia; 2 Rice Research Station, Louisiana State University Agricultural Center, Rayne, Louisiana, United States of America; 3 Genomics and Bioinformatics Research Unit, Agricultural Research Service, United States Department of Agriculture, Jamie Whitten Delta States Research Center, Stoneville, Mississippi, United States of America; 4 National Center for Genome Resources, Santa Fe, New Mexico, United States of America; 5 Plant Diversity Adaptation and Development Research Unit, Institut de Recherche pour le Développement, Montpellier, France; Oregon State University, UNITED STATES

## Abstract

Current advances in sequencing technologies and bioinformatics revealed the genomic background of rice, a staple food for the poor people, and provided the basis to develop large genomic variation databases for thousands of cultivars. Proper analysis of this massive resource is expected to give novel insights into the structure, function, and evolution of the rice genome, and to aid the development of rice varieties through marker assisted selection or genomic selection. In this work we present sequencing and bioinformatics analyses of 104 rice varieties belonging to the major subspecies of *Oryza sativa*. We identified repetitive elements and recurrent copy number variation covering about 200 Mbp of the rice genome. Genotyping of over 18 million polymorphic locations within *O. sativa* allowed us to reconstruct the individual haplotype patterns shaping the genomic background of elite varieties used by farmers throughout the Americas. Based on a reconstruction of the alleles for the gene *GBSSI*, we could identify novel genetic markers for selection of varieties with high amylose content. We expect that both the analysis methods and the genomic information described here would be of great use for the rice research community and for other groups carrying on similar sequencing efforts in other crops.

## Introduction

The advent of different gene array and sequencing technologies has made it feasible to conduct in-depth analysis of genome variation, population structure, pedigree relationships, and introgression in rice. Whole genome sequencing (WGS) has been recently carried on in pooled samples [1, 2], hundreds of individual accessions at low coverage [3, 4], dozens of accessions at higher coverage [5–8], and recently 3,000 accessions at about 15x coverage [9]. Not surprisingly, all these studies have detected a substantially greater number and diversity of variants compared to Single Nucleotide Polymorfism (SNP) assay methods [10–14], allowing an understanding of the genetic variability in rice with an unprecedented level of detail. Some of these studies have focused on revealing the domestication events leading to the development of *O. sativa* from its close wild relatives *O. rufipogon* and *O. nivara*. Classical molecular and population structure analysis suggests that japonica and indica varieties arose by independent domestication events followed by exchange of genetic material between the two subspecies [2, 8, 15, 16]. However, regions of extensive allele sharing between indica and japonica and coalescent simulations based first on sequenced gene fragments [17, 18] and later on WGS data [4] suggest that *O. sativa* developed from a single origin of *O. rufipogon* and that the indica varieties were later developed by large gene flow from wild to cultivated rice. Conserved regions across *O. sativa* are believed to be the product of selective sweeps fixating genes associated with traits such as shattering, erect growth, flowering time, and grain quality. Landraces and elite cultivars of both indica and japonica have also been genotyped, both with SNP array techniques and with sequencing data, and these data has been analyzed, first to understand the genetic diversity within *O. sativa* [11], later to identify individual introgressions related to agronomically interesting traits in elite cultivars [13], and finally to identify novel genes related to complex traits through Genome-wide Association Studies (GWAS) [10, 12, 14]. These and other studies produced significant advances in the understanding of the molecular basis of different agronomically important traits. Moreover, information produced by these new discoveries is being integrated in genomic databases to facilitate its use in both basic and applied genetics [19].

Different breeding programs are currently trying to take advantage of all this information for efficient development of improved varieties through molecular breeding techniques. In the case of marker assisted selection, effective marker design requires not only the genomic locations related to the trait of interest, but also the allelic variability within the cultivars that are being used by the breeding program [20]. Although previous sequencing efforts [4, 9] have generated information primarily from diverse, unadapted germplasm, rapid breeding advances will be based first on elite japonica or indica varieties already adapted to target environments. Therefore, improved varieties need to be sequenced to assess the extent of variability within adapted *O. sativa* germplasm at sequence resolution, to identify alleles that could be readily combined to drive rapid varietal improvement, and to prioritize low diversity regions requiring introgression of foreign alleles for further improvement.

The International Center for Tropical Agriculture (CIAT) and the RiceCAP project (http://www.uark.edu/ua/ricecap) initiated separate efforts to perform whole genome sequencing of elite germplasm that have been extensively used by breeders in Latin America and United States respectively. The fact that most of the elite lines of Latin America have an indica background and most of the U.S. elite lines have a tropical japonica background, enforced data sharing between these two initiatives enabling a comprehensive comparative genomic analysis of both groups of elite lines. This led to identify most of the genomic variation and admixture patterns shaping the genetic structure of the elite cultivars currently used by breeders in their specific environments. Accurate identification of subspecies-specific haplotypes was enforced combining publicly available sequencing data for 50 additional varieties [7], which includes not only accessions from indica and japonica cultivars, but also accessions from other groups within *O. sativa* such as aus and aromatic, and 10 wild relatives (5 *O. rufipogon* and 5 *O. nivara*). In this manuscript we describe the bioinformatic analysis that we carried out over extensive whole genome sequencing data to produce the comprehensive information resource on genomic variability described above, and discuss the use of this resource for further development of improved varieties through marker assisted selection.

## Materials and Methods

### Plant materials and accessions

We performed whole genome sequencing (WGS) of 21 elite cultivars from the CIAT rice breeding program and 33 elite cultivars from the United States rice breeding program (see S1 Table for details). These materials comprise a diverse representation of elite lines and commercial varieties from North and South America and Asia that exhibit desirable attributes for high grain yield, cooking quality, disease resistance, plant height, and maturity. Breeders consider these lines as highly relevant for rice improvement in the Americas. CIAT varieties include two advanced lines from IRGA (Instituto Rio Grandese do Arroz do Brasil), two from INIA-Uruguay (Instituto Nacional de Investigación Agropecuaria de Uruguay), five from Fedearroz (Federación Nacional de Arroceros de Colombia), one from INIA-Chile (Instituto de Investigaciones Agropecuarias, Chile) and one from Asoportuguesa (Asociación de Productores Rurales del Estado Portuguesa, Venezuela). Varieties from United States include one advanced line from IAC (Instituto Agronômico de Campinas, Brazil) and two from the Guanxi University of China. To relate our sequencing data with previous knowledge on rice variability, we reanalyzed WGS data publicly available at the NCBI SRA database for 50 accessions available at the International Rice Research Institute (IRRI), which were previously analyzed by [7]. These include 40 accessions distributed among the major populations of *O. sativa* and 10 varieties from the close wild relatives *O. rufipogon* and *O. nivara*.

### DNA sequencing

Each variety from the CIAT collection was planted in the greenhouse facility at CIAT. Genomic DNA was prepared from a single plant as follows: 1 g of leaf tissue of a 45-DAP seedling was collected and ground with liquid nitrogen. DNA was isolated according to the urea-phenol extraction protocol modified from [21]. DNA quality was tested before whole-genome sequencing so that the concentration exceeded 500*ng*/*μL* and the A260/280 ratio was 1.8. DNA was sequenced on the Illumina HiSeq 2000 by the Yale Center for Genome Analysis (http://medicine.yale.edu/keck/ycga/index.aspx). DNA from the U.S. accessions was isolated and prepared for sequencing as described by [22]. All sequencing data generated for this work are available at public repositories (see Data Availability statement for details). Additional sources for bulk data access include Gramene (ftp://ftp.gramene.org/pub/gramene/release45/data/vcf/oryza_sativa/Duitama/), the data store module of iPlant [23], and the European Variation Archive (EVA) [24]. We are working with major online rice genomics data resources such as Gramene [25] and the Rice Annotation Project [26] to provide the annotations and variation data for rice researchers from their rice genome browser.

### Mapping and variant calling

We downloaded the reference genome IRGSP-1.0 [27] from the Rice Genome Annotation Project web page (http://rice.plantbiology.msu.edu/), including their corresponding GFF3 file with gene functional annotations. We used the NGSEP pipeline [28] to align reads to the reference and discover SNPs, indels, repeats and Copy Number Variants (CNVs). NGSEP uses bowtie2-2.1.0 [29] for read alignment, which we ran with default parameters, except for the maximum number of alignments per read, which we set to 3, and the minimum and maximum fragment length for valid paired-end alignments, which we estimated separately for each variety aligning their first 250000 fragments and then plotting the distribution of estimated insert lengths (Script available at the NGSEP web site http://sourceforge.net/projects/ngsep/files/Library/scripts/). We used the recommended parameters of NGSEP for analysis of WGS data: 1) Minimum genotype quality 40; 2) Maximum value allowed for a base quality score 30; and 3) Maximum number of alignments allowed to start at the same reference site 2. We set the prior heterozygosity rate (h option) to 0.0001 to give a larger prior probability to homozygous genotypes. We also used NGSEP for functional annotation of variants, filtering, and conversion from VCF to other formats for further downstream analysis. Flapjack software [30] was used for visualization of SNP genotypes across the samples. To identify characteristic CNVs for a population we used the following procedure: given two populations *P*_1_ and *P*_2_ and a CNV that is identified in *x*_1_% of *P*_1_ and *x*_2_% of *P*_2_ with an average number of copies *n*_1_ and *n*_2_ for *P*_1_ and *P*_2_ respectively, we call such CNV characteristic for *P*_1_ relative to *P*_2_ if *x*_1_ − *x*_2_ > 50% or both *x*_1_ > 50% and *n*_1_ − *n*_2_ > 2.

We ran mrCaNaVaR [31] to compare their predicted CNVs with those predicted by NGSEP. To make the results comparable, we took as input for mrCaNaVaR the alignments provided by bowtie2 and we set a long window span of 500bp and a short window span of 100 bp. We did not mask the repetitive regions in the reference genome to allow mrCaNaVaR to predict CNVs in such regions.

### Diversity and population structure

We used the neighbor joining algorithm implemented in SplitsTree4 [32] for construction of distance-based unrooted dendograms. To obtain confidence values we performed 1,000 replicates of the bootstraping analysis available in SplitsTree4. Dendograms with bootstraping confidence values and branch lengths are available as supplementary material (S1–S4 Files). We also used SplitsTree4 for visualization of the dendograms. For population analysis we used the individual-based Bayesian clustering method implemented in STRUCTURE v.2.3.4 [33]. We assumed the admixture model with correlation of allele frequencies and we varied the number of populations (K) from 1 to 8. The length of the burn-in period was set on 10,000 and the number of Markov Chain Montecarlo (MCMC) Reps after burn-in on 20,000. To estimate diversity across the genome, we developed a custom java script that calculates the number of nucleotide changes between each pair of accessions either within a window or within a gene, and we calculated the average number of pairwise distances for each subpopulation and between user-defined pairs of subpopulations as suggested by [7]. Genome-wide plots of diversity were developed using CIRCOS v.0.66 [34]. To calculate the LD-Decay of indica, japonica and overall, we selected high quality SNPs with minor allele frequency (MAF) above 0.1 and then we ran PLINK [35] using a maximum window of 2Mbp for pairwise calculation of *r* ^2^ values.

### Admixture analysis

We built a custom java script to identify SNPs segregating for at least one of the main seven Oryza populations (*O. rufipogon*, *O. nivara*, aus, aromatic, indica, tropical japonica, temperate japonica). The script takes as input a VCF file, calculates the allele frequency of the reference allele for each population and retains SNPs in which the absolute difference in allele frequencies for two populations is at least 0.6. The script also produces an output VCF file with one additional sample column for each subpopulation. This column contains a genotype representing the most frequent allele within each subpopulation or a heterozygous genotype if the MAF within the population is greater than 0.4. We built a second script that takes as input this VCF file and calculates for each variety and each non-overlapping window of 50 SNPs its closest population assignment using a simple algorithm described in [36]. In brief, for a given pair of genotype calls over 50 SNPs, for each SNP the script adds 1 if at least one of the two genotypes is heterozygous, 2 if the two genotypes coincide, and −2 if the two genotypes are homozygous and different. Hence, a maximum score of 100 will be obtained for a pair of genotype calls over a window of 50 SNPs if and only if they are equal and do not contain any missing or heterozygous call. For each variety and each window the script performs a unique population assignment calculating the score between the genotype calls of the variety against the genotype calls of each of the seven populations. A population will be assigned for a variety within a window if there are at least 40 SNPs genotyped and the score is at least 50. This assignment will be considered unique if the differece between the best and the second score is at least 10. If a unique assignment cannot be made, the script outputs the names and the scores of the two populations ranked first and second. Finally, the script also compares the population genotypes against themselves to identify windows difficult to discriminate due to conservation between subpopulations.

### Screening and SNP genotyping for amylose content

A total of 47 elite indica rice accessions were genotyped using the Fluidigm technology (EP1TM system) based on SNPtype assays and allele-specific PCR. Screening the same varieties for amylose content (AC) was carried out in five plants per accession. AC was determined using a near-infrared spectroscopy (NIRSystems 6500®) [37]. To assess significance of the differences between AC for the six haplotypes identified within *GBSSI*, analysis of variance was performed using SAS version 9.2 (SAS Institute Inc., Cary, NC, USA) using a significance level of 0.05, adjusted with the Bonferroni correction [38].

## Results

### Whole genome sequencing of elite rice cultivars

Collaboration between independent sequencing efforts combined with availability of data from previous works in public databases allowed us to perform an integrated analysis of whole genome sequencing (WGS) reads for 94 *O. sativa* varieties and 10 wild relatives (see Methods and S1 Table for details of the sequenced accessions). The whole dataset includes 3.8 billion reads and 699 Gbp of raw data. The initial average coverage per sample ranged between 2.87x and 64.83x. Except for the 13 varieties initially sequenced by the RiceCAP project [22], all other cultivars were sequenced at over 8x average coverage. We could align over 90% of the reads to the Nipponbare reference genome for most of the *O. sativa* accessions and over 80% of the reads for the *O. rufipogon* and *O. nivara* accessions (S2 Table). We ran the NGSEP pipeline [28] to identify Single Nucleotide Polymorfisms (SNPs), indels, repeats, and Copy Number Variation (CNVs) on the 104 sequenced samples. We identified over 23 million polymorphic sites in the whole dataset and we genotyped each of the 104 accessions on these sites. Over 80% of them fall within repeat elements (see next section for details). Table 1 shows the number of SNPs obtained using different filtering strategies that we applied to perform the different types of analysis carried out in this study. From the 4.4 million SNPs found outside repetitive regions, we could genotype over 95% in at least 50 accessions (S1 Fig). We verified that with an average coverage above 10X, we could genotype over 80% of these SNPs in most of accessions, although this percentage was reduced substantially as coverage reduces (S1 Fig). If only the *O. Sativa* accessions were considered, the number of polymorphic sites in non-repetitive regions was reduced to three million with further reductions observed only if indica or japonica accessions were evaluated (Table 1). Approximately 13% of the selected SNPs were located in coding (non intronic) regions no matter which subpopulation was considered. For each filtering strategy and each subpopulation, we calculated average dN/dS ratios over transcripts with at least one synonymous mutation and we found that, as filters become more stringent, dN/dS values consistently reduce from 0.57 to 0.26. This outcome can be explained not only by the increase in genotyping specificity obtained after applying the different filters, but also by the fact that after removing variants within repeat elements or recurrent copy number variation events, the single copy genes affected by the remaining variants tend to be more conserved to prevent complete loss of function. Only up to 1.5% of the SNPs outside repeat regions produced a stop codon and this percentage was decreased as more stringent filters were applied. As expected, more than 60% of the SNPs in the entire dataset exhibited minor allele frequencies (MAFs) below 0.05 because they were only polymorphic within the 10 *O. rufipogon* and *O. nivara* accessions (S2 Fig). Rare alleles were also predominant within *O. Sativa*, but the percentage decreased to 45% (compared to the percentage obtained including wild relatives). Within the indica subpopulation, SNPs with MAF between 0.05 and 0.15 were more common than SNPs with MAF below 0.05. Finally, about 50% of the SNPs within japonica showed MAF below 0.05, mainly due to the small representation of temperate japonica compared with tropical japonica and the lower overall diversity within japonica.

**Table 1:** SNPs among 104 rice cultivars.

		No filter	Filter 1	Filter 2	HQ (Filter 3)
All samples	Total	23,389,776	4,416,199	669,874	84,578
	Synonymous	2,016,496	241,765	37,300	6,516
	Missense	2,419,534	332,340	40,950	6,082
	Nonsense	137,681	9,659	777	76
	% coding	19.55%	13.22%	11.80%	14.98%
	dN/dS	0.56	0.53	0.38	0.27
*O. sativa*	Total	18,572,995	3,027,636	671,175	106,193
	Synonymous	1,711,097	167,483	37,514	7,273
	Missense	1,987,782	236,975	42,758	7,358
	Nonsense	109,757	6,873	843	109
	% coding	20.51%	13.59%	12.09%	13.88%
	dN/dS	0.57	0.53	0.39	0.29
Indica	Total	11,158,840	1,696,132	870,257	208,384
	Synonymous	1,124,730	97,690	49,166	14,947
	Missense	1,180,787	134,316	60,396	15,367
	Nonsense	61,229	3,733	1,475	257
	% coding	21.21%	13.90%	12.76%	14.67%
	dN/dS	0.53	0.45	0.39	0.28
Japonica	Total	9,220,167	1,587,839	544,560	127,419
	Synonymous	831,744	92,171	32,404	8,551
	Missense	956,359	128,613	39,855	9,108
	Nonsense	48,153	3,516	857	162
	% coding	19.92%	14.13%	13.43%	13.99%
	dN/dS	0.55	0.47	0.36	0.26

SNPs obtained for the 104 varieties analyzed in this study the subset of varieties belonging to the *O. sativa* species (removing the 10 *O. rufipogon* and *O. nivara* wild relatives), the varieties clustered within the indica subtree, and the varieties clustered within the japonica subtree. The last three columns show the number of SNPs retained after applying three progressive filters: 1) Remove SNPs within identified repetitive regions in Nipponbare, 2) Remove singleton SNPs (e.g. with the minor allele present in only one variety) and SNPs in regions in which at least three varieties report copy number variation, and 3) Remove SNPs in which at least one variety reports copy number variation, SNPs located less than 10 bp away from any other variant, and SNPs with less than 80 individuals genotyped.

**Fig 1:**
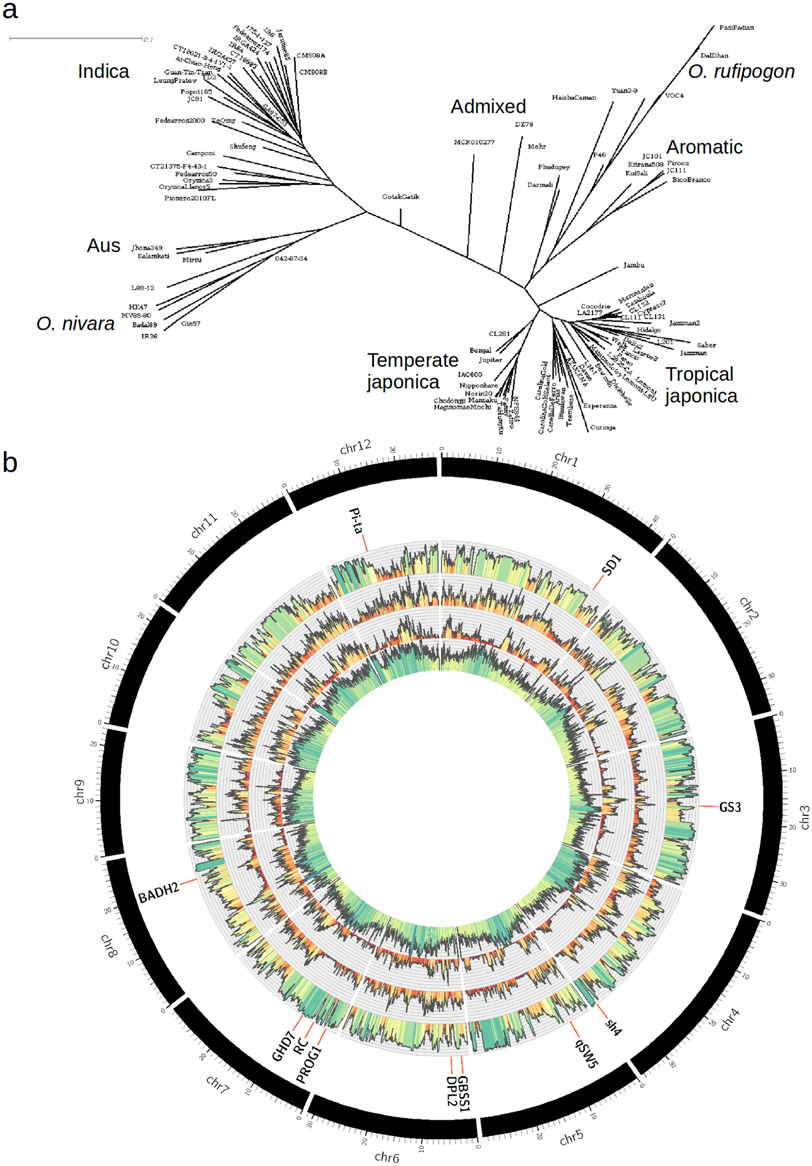
Genome-wide diversity patterns for sequenced cultivars of indica and japonica. a) Neighbor joining dendogram for the full dataset of accessions analyzed in this study. b) Moving from within to outside, the circles have the following information: 1). Density of repeat elements (0% to 100%). 2) Diversity within japonica (0–10). 3) Diversity within indica (0–10). 4) Pairwise Fst between indica and japonica (0–1). For each population, diversity is estimated in 100kbp windows as the average number of pairwise differences per kilobasepair (See methods for details). Green colors indicate values close to the maximum on each category (or larger for the case of diversity values). Red colors indicate values close to zero. Yellow colors indicate intermediate values. Genomic locations of genes related to selective sweeps are shown in red lines.

For further validation of our genotype calls, we built neighbor-joining dendograms using the genetic distances estimated from the high quality SNPs (filter 3 in Table 1) identified in the whole dataset and within each subpopulation (Fig 1a, S3 Fig and S1–S4 Files). The dendograms were consistent with those shown in previous studies [4, 7]. Nonetheless, we obtained a clearer separation between indica and *O. nivara* accessions when compared with [7] presumably due to greater number of indica accessions included in our analysis. Population structure analysis of the high quality SNPs within *O. sativa* accessions consistently separated the indica, aus, aromatic, temperate japonica and tropical japonica populations as values of the number of allowed populations increased from 2 to 5 (S4 Fig). Pairwise Fst values predicted by structure [33] ranged from 0.1 for tropical vs. temperate japonica to 0.37 for indica vs temperate japonica. These pairwise Fsts were smaller than previously reported [4] probably because the elite lines in our study contributed large haplotypes of outgroup introgressions that reduced the overall segregation between indica and japonica. We calculated for each population and for each filtering strategy the number of private SNPs (polymorphic in only one population) (S5 Fig) and we found that the groups indica and *O. rufipogon* showed the largest number of private SNPs and that aromatic and temperate japonica showed the smallest numbers of private SNPs. We finally calculated the linkage disequilibrium (LD) decay for *O. sativa*, indica and japonica and we found that, consistent with previous studies [3, 14], the LD-decay was faster for indica compared to japonica and to *O. sativa* (S6 Fig).

To compare diversity within and between indica and japonica across the genome, we calculated the average number of pairwise SNP differences without filters over 100 kbp (kilobasepair) windows. We selected accessions with coverage greater than 10x clearly clustering within the indica or the japonica groups (not including aus and aromatic), and we estimated diversity within indica, within japonica, between indica and japonica, and for the whole group. Diversity values (pairwise differences per kbp) were on average 2.58 within indica, 1.96 within japonica, 5.9 between indica and japonica, and 3.93 overall. These values were about two times larger than those reported by [4] for low coverage sequencing (below 2x), but were relatively consistent with those reported by [7] for the subset of 50 accessions with over 10x coverage also included in this study. Having low coverage per sample reduces the percentage of genotyped sites which consequently reduces the number of differences identified for each pair of samples. The overall Fst between indica and japonica, estimated from these averages as one minus the proportion of the diversity within groups relative to the diversity between groups [7], was 0.64. Although the number of indica accessions included in this analysis (23) was smaller than the number of japonica samples (38), the indica population showed greater overall diversity than japonica, which is consistent with previous studies [4, 11, 14]. However, this pattern of diversity is not consistent across the whole genome. Fig 1b shows the array of diversity for japonica and indica, and the Fst between indica and japonica. Large regions of almost complete differentiation (Fst close to 1) between indica and japonica are observed in every chromosome. However, in most of the genes known to be related to domestication traits the differentiation between indica and japonica and the diversity of both populations is reduced. The longest region of conservation for both populations is located between 10 and 15Mbp of chromosome 5. Indica-specific conserved regions can also be observed in chromosome 7, 8, and the start of chromosome 9. We calculated diversity values for the indica population within the selective sweeps reported by [4] for indica (S7 Fig) and we found that the average diversity in these regions reduced to 1.44 for indica becoming almost equal to the estimate for japonica (1.46). Moreover, we found that 48 of the 60 selective sweeps contain windows in which the diversity within indica was below 1.

### Repetitive elements and novel structural variation

Merging results from independent analysis of each sequenced variety, we developed a catalog of 164,372 repetitive regions covering 176.9 Mbp (roughly 45%) of the rice genome. As expected, centromeres and telomeres showed a high density of repeat elements (Fig 1b). Repeat density was also high in known large duplication events such as the starts (first two Mbp) of chromosomes 11 and 12 [39]. We compared the repeats identified using NGSEP with a catalog of annotated repeat elements generated by Rod Wing at The University of Arizona (personal communication). We found that about 80% of the DNA identified as repetitive by NGSEP was annotated as a repeat element and that 78% of the annotated elements were covered by NGSEP repeat regions. A majority (98%) of the annotated repeats that NGSEP could not identify have lengths below 500bp. This is expected because reads can be aligned uniquely to short sparsed repeats taking advantage of the paired-end information. The longest region not identified by NGSEP is a 6.2Kbp region on chromosome 6 covering the retrotransposon LOC_Os06g50200. A blast search of this region back to the reference genome shows that the second best hit only has an alignment length of about 1,924 bp and an identity of 71.73%, which means that although this retrotransposon is a member of a repeat family, it has accumulated enough mutations to be considered a unique sequence for alignment purposes. Similar analysis of other three missed repeats with lengths above 5,000 bp yielded the same outcome. Nevertheless, following a conservative approach for the downstream analysis, we merged the annotated elements with the repeats identified by NGSEP to produce a unified annotation of repeats in the reference genome (hereafter referred to as repeats). We used this unified dataset for characterization of other types of variants. We further compared these repeat regions with the sequences masked as repeats in the version of the reference genome available in the RAP-DB web page (http://rapdb.dna.affrc.go.jp/) and we found that 154.3Mbp (96.65%) of the 159.6Mbp masked by RAP-DB are covered by the regions described above.

**Fig 2:**
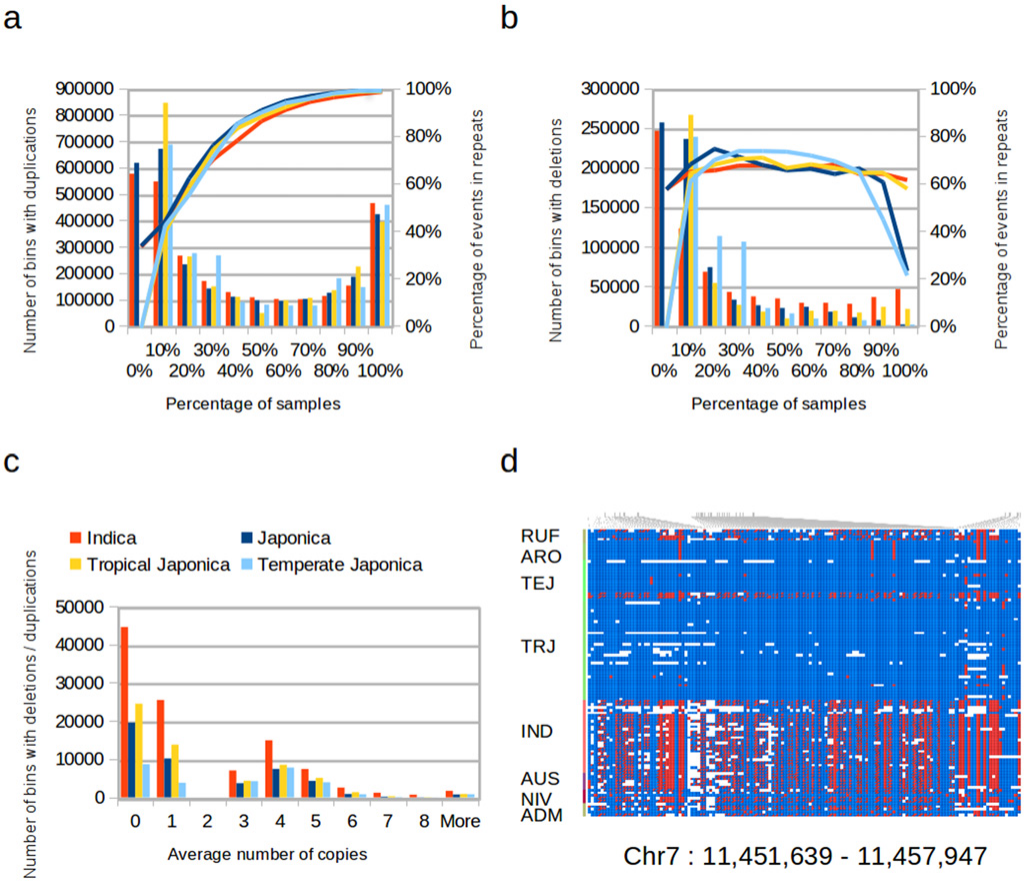
Comparison of CNV calls in rice cultivars. Number of 100bp bins with a) duplications, and b) deletions discriminated by the percentage of each population in which the event is reported (red: Indica, blue: japonica overall, yellow: tropical japonica, and light blue: temperate japonica). The lines indicate the percentage of bins for each category falling within repetitive regions in Nipponbare. c) Number of bins not spanning Nipponbare repeats with predicted CNVs common for each subpopulation (indica, japonica, tropical japonica, and temperate japonica) discriminated by the predicted copy number, being two the normal copy number for a diploid region. d) Example of a discriminative duplication between indica and japonica. Reads taken from the two copies of this region present in indica samples align to the same genomic location producing clusters of heterozygous SNPs. Colors in the left panel differentiate the following groups: *O. rufipogon* (RUF), aromatic (ARO), temperate japonica (TEJ), tropical japonica (TRJ), indica (IND), aus (AUS), *O. nivara* (NIV), and admixed (ADM). Homozygous genotype calls carrying the reference allele are colored blue. Homozygous genotype calls carrying an allele different from the reference are colored red. Heterozygous genotype calls are colored half blue and half red.

We performed on each sample the read-depth analysis provided by NGSEP to identify regions with copy number variation (CNVs). For this analysis we discarded 29 accessions for which the read-depth distribution suggested that coverage was not evenly distributed along the genome (S2 Table). We compared the CNVs identified for 21 indica, 12 temperate japonica, and 18 tropical japonica varieties, which were chosen following the clusters observed in the distance trees. To facilitate comparisons among samples and events with variable lengths, we retrieved and compared the copy number estimation for each sample on non-overlapping bins of 100bp across the genome. For each group we identified between 2.3 and 2.8 million bins with duplication events and between 475 and 725 thousand bins with deletion events. This represents over 10 times more variation than that observed using high-density array comparative genomic hybridization [40] or using the read-depth analysis carried on by [7] for 50 accessions. Figs 2a and 2b shows the distribution of bins with duplication and deletion events as a function of the percentage of samples in which the variation was discovered. Between 55% and 65% of the bins with duplications and between 70% and 95% of the bins with deletions were reported by less than half of the samples within each subpopulation. We also found that most of the bins with duplications (over 97% for common duplications) overlap with repeats. In contrast, only 65% of the bins with predicted deletion events overlap with repeats. After removing bins within repeats and bins with events reported in less than half of the samples within each population, the number of bins with CNVs was reduced to 105,606 for indica, 58,896 for tropical japonica and 30,158 for temperate japonica. This is expected because most of the common duplications within the temperate japonica accessions in our study should already be identified as repeats in the Nipponbare reference sequence which is also temperate japonica. Likewise, common deletions within temperate japonica should mostly correspond with DNA present in Nipponbare and absent in other temperate japonica cultivars. Consequently, filtering out repetitive regions, recurrent duplications are more common than recurrent deletions within temperate japonica, whereas recurrent deletions are more common than recurrent duplications within tropical japonica and within indica. Fig 2c shows the distribution of bins with common CNVs in non-repetitive regions for different average numbers of copies. For every population, homozygous deletions were twice more common than heterozygous (copy number 1) deletions. Moreover, homozygous duplications (copy number 4) were twice more common than heterozygous duplications (copy number 3). Although we do not have a gold-standard set of CNVs to perform a systematic comparison with other methods, we performed an initial comparison of the CNVs identified using NGSEP with the CNVs identified using mrCaNaVaR [31]. On average MrCaNaVaR called deletions on about 70 Mbp for each variety, which is close to 4 times more genomic sites for indica and close to 6 times more sites for japonica compared to NGSEP (S8 Fig). MrCaNaVaR also called 1.5 more regions as duplications for both indica and japonica varieties compared to NGSEP. For most of the samples over 80% of the deletions and over 70% of the duplications identified by NGSEP were also identified by mrCaNaVaR, which provides additional confidence on the events called by NGSEP.

Finally, we performed pairwise comparisons among the indica, tropical japonica, and temperate japonica populations to identify CNVs characteristic of a particular population (See methods for details). We found over two times more bins with characteristic CNVs for indica relative to temperate japonica or to tropical japonica than the opposite (S3 and S4 Tables). We also detected three times more bins with characteristic CNVs for indica relative to temperate japonica than characteristic CNVs for tropical japonica relative to temperate japonica. Consistent with the percentages observed within each population, ∼ 64% of the bins within characteristic deletions and ∼ 96% of the bins within characteristic duplications overlap repeat regions. Fig 2d shows the SNPs identified in a characteristic indica duplication relative to japonica. Most of the SNPs in this region appeared as heterozygous in the indica varieties because the reads that were sequenced from different copies of indica cultivars align to the single copy present in Nipponbare and differences between copies were identified as clusters of heterozygous SNPs.

### Diversity and haplotype patterns in agronomically important genes

Elite cultivars have been developed over the last decades by breeders looking for introgression of specific alleles to improve desirable traits such as high yield, grain quality or resistance to abiotic and biotic stresses. Starting from the 670 thousand non-singleton SNPs in non-repetitive regions and regions with up to two CNVs (Filter 2 in Table 1), we selected 329,819 SNPs that segregated in at least one of the seven identified populations (aus, aromatic, indica, *O. nivara*, *O. rufipogon*, temperate japonica and tropical japonica). After identifying the most frequent allele of each SNP within each population, we selected non-overlapping windows of 50 SNPs and calculated for each accession its most likely population origin for each window based on its observed haplotype pattern (See Methods section for details). For most of the varieties sequenced at 10x or more, this analysis identified unique population assignments for at least 100 Mbp. As expected, characteristic haplotypes of temperate and tropical japonica were the most difficult to differentiate. The complete table of population assignments for the 104 varieties is included as a supplementary material (S5 Table).

**Fig 3:**
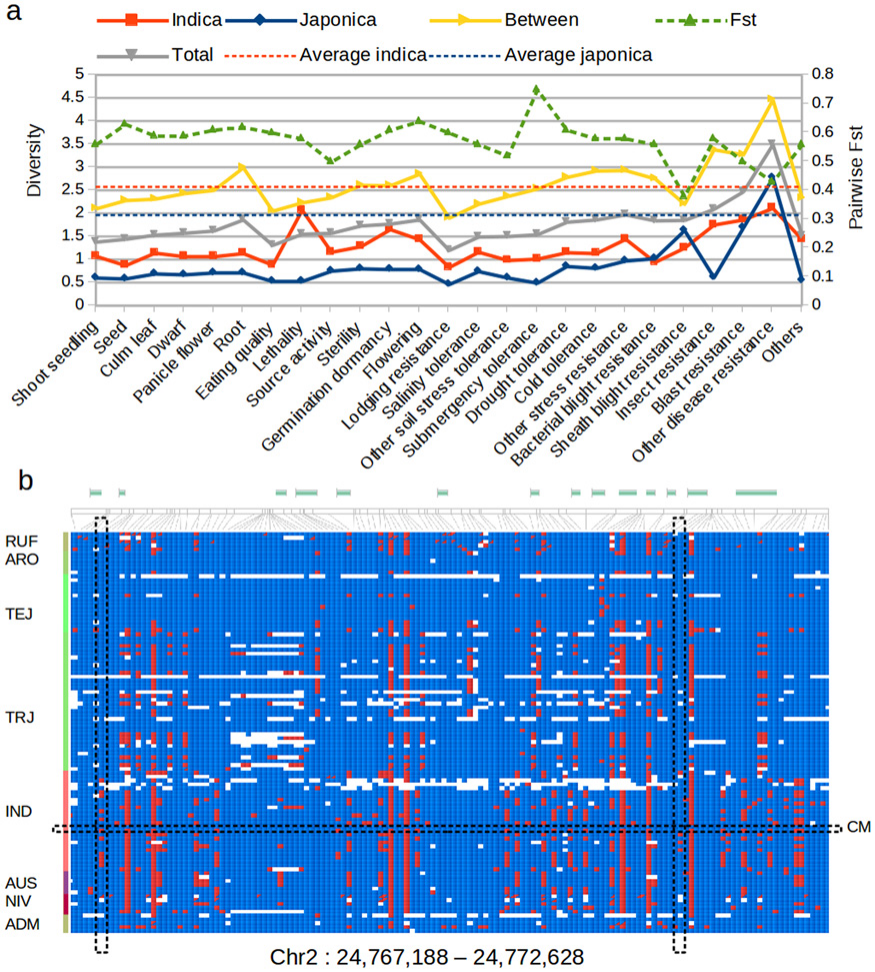
Diversity within agronomically important genes. a) Average diversity (average number of pairwise differences per kilobasepair) for genes grouped following the minor categories defined in the OGRO database. Red and blue dashed lines indicate average diversity values across the whole genome (including non-genic regions) within indica and within japonica respectively. The green dashed line, showing the average pairwise Fst between indica and japonica, is scaled in the secondary Y axis. b) SNPs identified within the gene LOC_Os02g40860 in the 104 varieties analyzed in this study. Characteristic alleles of the temperate japonica haplotype are painted blue. Vertical dashed rectangles show the locations of the two missense SNPs with high minor allele frequency within indica. The horizontal rectangle shows the haplotype of the variety Camponi. Colors in the left panel differentiate the following groups: *O. rufipogon* (RUF), aromatic (ARO), temperate japonica (TEJ), tropical japonica (TRJ), indica (IND), aus (AUS), *O. nivara* (NIV), and admixed (ADM).

To assess potential functional effects of the observed patterns of admixture, we selected the SNPs within or close to genes that have been identified as related to agronomically important traits and summarized in the OGRO database [19]. Fig 3a shows the average diversity values for the genes included in the 25 minor trait categories proposed by [19]. As expected, average diversity values within coding regions were lower than averages for the whole genome both within indica and japonica. Peaks of high diversity within indica were observed for genes related to lethality, insect resistance, blast resistance, and other disease resistance. Within japonica large diversity was observed for resistance to diseases such as sheath blight caused by Rhizoctonia solani, blast caused by Magnaporthe grisea and other diseases. These patterns were generally consistent with traits prioritized by CIAT and LSU in their breeding programs.

The peak observed for lethality (Fig 3a) is partly explained by the fact that only two genes are included in this category (LOC_Os02g40860 and LOC_Os04g38950). The gene LOC_Os02g40860 is a member of the casein kinase I family and it has been related to hybrid weakness and growth retardation [41]. Allelic variation in our dataset shows three more or less differentiated haplotype patters for temperate japonica, tropical japonica and indica, as well as a large number of low frequency SNPs (Fig 3b). Part of the increased variability within tropical japonica and within indica is produced by introgression of haplotypes characteristic from temperate japonica identified in the tropical japonica cultivars Carolina-Gold, Carolina-Gold-Select, LM-1, Arias, Binulawan, Canella-de-Ferro, Curinga, Esperanza and Parao, as well as in the indica cultivar Camponi. Although eight missense SNPs were identified for LOC_Os02g40860, six of them had their minor allele present in only one or two varieties. Two missense SNPs in exons 2 and 13 showed relatively high MAF within indica and hence could be potentially useful to track indica-specific alleles of this gene. Conversely, the introgression observed in Camponi should facilitate interspecific crosses of this cultivar with temperate japonica lines (compared with other indicas) because the effect of autoimmunity observed by [41] should not be observed in this case. The gene LOC_Os04g38950 is a glutamine amidotransferase which has been shown to produce dwarfing, narrow leaves, short roots and abnormal flowers if silenced [42]. Our data shows almost complete conservation of this gene within japonica and an indica-specific haplotype pattern composed by 34 SNPs (S9 Fig). Three of these SNPs located in exons 1 and 2 produce changes in the aminoacid sequence. Most of the variability observed in this region within indica is explained by the introgression of the japonica haplotype in the varieties Ai-Chiao-Hong, Guan-Yin-Tsan, Leungpratew, IR8, Camponi, CT21375, Fedearroz50 and Oryzica 3.

### Novel SNP markers for amylose content

**Fig 4:**
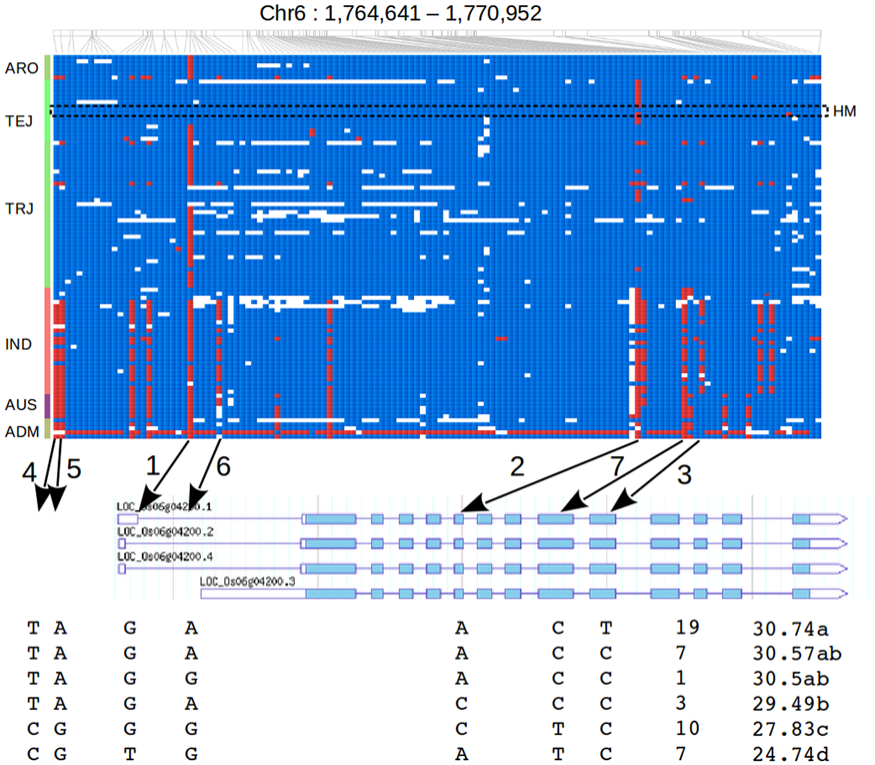
Observed haplotype patterns within the gene *GBSSI* for *O. sativa* accessions. Alleles of the temperate japonica variety Haginomae Mochi (dashed rectangle), which has the defective alleles for Waxy-1 to Waxy-7, are colored blue. Arrows indicate locations within the gene for the discriminative SNPs Waxy-1 to Waxy-7. Colors in the left panel differentiate the following groups: aromatic (ARO), temperate japonica (TEJ), tropical japonica (TRJ), indica (IND), aus (AUS) and admixed (ADM). The table below shows the six haplotype configurations in an independent group of 48 indica elite varieties with variable amylose contents. The last two columns are the number of samples showing the haplotype and the average amylose content for each haplotype. Characters a, b, c and d in the last column differentiate haplotypes with significant differences in amylose content.

For breeding purposes, one of the main goals of performing sequencing of elite cultivars is the identification of markers that could be used for marker assisted selection. Bearing this in mind, we investigated the variation observed within the gene *GBSSI*, located at 1.76Mbp of chromosome 6, which is known to be related to amylose content [43]. We identified a total of 112 SNPs close to this gene, 82 of them only variable in the admixed variety HaishaCaman (Fig 4). From the remaining 30 SNPs, the minor allele of 17 was carried by at least four varieties. Three of these SNPs (termed Waxy-1, Waxy-2, and Waxy-3) were previously reported as markers for amylose content [44, 45]. Waxy-1 is located in the first splicing site of one of the transcripts identified for *GBSSI* which probably blocks transcription of this isoform. In this case the defective allele is the minor allele in our population and it is mostly present in temperate japonica accessions. Waxy-2 and Waxy-3 produce single amino acid changes in exons 6 and 10 respectively. For both markers, the advantageous allele is more frequent in indica than in japonica, although the advantageous allele of Waxy-2 is also frequent in temperate japonica. Besides these markers, we selected four additional SNPs with the minor allele present mostly in indica cultivars and we termed them Waxy-4, Waxy-5, Waxy-6 and Waxy-7. Waxy-4 and Waxy-5 are located about 800 bp before the transcription start site. Waxy-6 is located within the first intron, and Waxy-7 is a synonymous SNP in exon 9.

We genotyped these seven markers in a population of 47 indica accessions with variable amylose content using the Fluidigm chip technology. Consistent with previous studies [43], we found that the haplotype built with the advantageous alleles of Waxy-1, Waxy-2 and Waxy-3, and the indica allele of Waxy-4, Waxy-5, Waxy-6 and Waxy-7 showed the highest amylose contents. Moreover, the indica allele of Waxy-4, Waxy-5, and Waxy-7 significantly differentiates varieties with high and low amylose content. We tested each marker independently for relationship with the trait and we found that, except for Waxy-2, all markers were significantly related to amylose content. Waxy-4 and Waxy-5 are in complete linkage disequilibrium (LD) and they are also in almost complete LD with Waxy-6 and Waxy-7, although a few recombinants between these markers were observed in the sequenced varieties. These results suggest that either Waxy-4, Waxy-5, or another variant upstream of *GBSSI* and in high LD with Waxy-4 and Waxy-5 spans a cis-regulatory region of *GBSSI* and hence plays a role in the transcription efficiency of this gene.

## Discussion

Genetic improvement of cultivated plants for increased yield in different environments is one of the most important strategies for significant improvement in food production and hunger alleviation in a rapidly growing human population [20]. The use of high throughput sequencing technologies will generate nearly complete genomic information of entire genebanks, which will boost the development of improved varieties by breeding programs using molecular techniques [9]. Bearing this in mind, sequencing efforts were initiated at CIAT, the USDA RiceCAP program, the USDA-ARS Genomics and Bioinformatics Research Unit, Stoneville, MS, and at LSU to obtain detailed genomic information for elite cultivars largely used by farmers throughout the Americas. Collaborative bioinformatic analysis of the sequencing data in conjunction with publicly available datasets provided us an in-depth understanding of our elite germplasm allowing identification of the haplotype patterns shaping the genetic architecture of each cultivar. To validate the accuracy of our SNP calls, we performed different standard population genomics analyses to reveal the overall population structure and the array of diversity across the genome, within and between indica and japonica, and we obtained results that were generally consistent with previous studies [4, 7, 11, 14]. Further bioinformatic analysis revealed novel structural variation and variety-specific admixture patterns. Moreover, because whole genome sequencing (WGS) of inbred rice cultivars enables direct reconstruction of the alleles carried by each variety in nearly every gene, we were able to efficiently and accurately determine and compare the variability within elite cultivars for hundreds of genes known to be related to important agronomic traits [19]. We expect to use the SNPs identified in these genes for rapid development of improved rice varieties for both Latin America and the United States.

In our experience with marker assisted selection (MAS), information of structural variation has been critical to refine strategies for designing genetic markers that can be effectively used to track desired alleles. Generic markers designed from variation on global germplasm can become ineffective for specific crosses if they overlap with presence/absence variation in parental lines. Recent duplications can also confound the genotype calls obtained with generic markers. Unfortunately, current reports of structural variation in rice have been limited to few varieties and only a few mega base-pairs [7, 40]. Combining different algorithms for discovery of structural variants from WGS data, we comprehensively identified regions with subspecies-specific structural variation as well as variety-specific events. Although the algorithms designed so far for discovery of structural variants from WGS data are generally not as accurate as algorithms for SNP discovery [28, 46], cross comparison of the variation discovered within samples sequenced at different facilities and at different average coverages allowed us to validate a large percentage of the CNVs and large deletions identified by our pipeline and to determine the minimum coverage and desired distribution for accurate discovery of structural variants. We integrated this information into our pipeline for markers design to increase the genotyping success rate of the SNPs that we are currently designing for different MAS experiments.

Given the importance of high amylose content as a component to achieve the grain quality required by Latin American and U.S. markets, we investigated the variability present in our sequenced germplasm within the gene *GBSSI*, which is known to be related to amylose content [43]. Besides confirming the three SNP markers previously identified within this gene [44, 45] we developed four novel SNP markers and we showed through genotyping of an independent population that these markers can be used to track alleles conferring high levels of amylose content in rice grains. These promising results encouraged us to follow the same general strategy to develop markers for other traits such as cold tolerance, resistance to blast and viruses, and yield components. We have currently designed close to 400 SNP markers for MAS, achieving a genotyping success rate close to 95%. The availability of WGS data provides us the marker density required to perform simultaneous tracking of alleles of different genes either conferring different desired characteristics or with additive or epistatic effects for a single trait. Moreover, WGS data on adapted elite lines enables the design of custom markers that ensure keeping the genomic background of varieties with high yield. The variant density achieved with WGS allows the design of flanking markers for each target gene which we can use to track recombinations between donor and background haplotypes at very short distances from the target genes, alleviating the potential effect of linkage drag during introgression of foreign alleles [20, 47]. We believe that both the analysis pipelines and the genomic variation described in this manuscript will be of great use for other groups looking for genetic improvement of rice and even for similar efforts in other crops.

## Supporting Information

S1 Fig SNP genotyping statistics. A) Number of SNPs obtained in non-repetitive regions (filter 1 in Table 1) for different minimum number of individuals genotyped. B) Percentage of SNPs genotyped as a function of the average coverage obtained from reads aligned to the Nipponbare reference genome.
(TIF)

S2 Fig MAF distributions. Distribution of allele frequencies for the SNPs found in non-repetitive regions (filter1 in Table 1) for the 104 varieties analyzed in this study (yellow bars) and for selected subsets based on membership to the *O. sativa* species (green), or membership to the two major subspecies within *O. sativa*, indica (red) and japonica (blue).
(TIF)

S3 Fig Dendograms for *O. sativa* varieties. Neighbor joining dendograms for A) the 94 *O. sativa* varieties, B) indica varieties, and C) japonica varieties.
(TIF)

S4 Fig Population structure clustering of the *O. sativa* accessions. Clusters obtained with the Structure software changing the number of allowed populations (k parameter) from 2 to 5.
(TIF)

S5 Fig Private SNPs. Number of SNPs polymorphic only within one population for the seven analyzed populations and for the following filtering strategies: 1) No filters 2) Remove SNPs within identified repetitive regions in Nipponbare, 3) Remove singleton SNPs (e.g. with the minor allele present in only one variety) and SNPs in regions in which at least three varieties report copy number variation, and 4) Remove SNPs in which at least one variety reports copy number variation, SNPs located less than 10 bp away from any other variant, and SNPs with less than 80 individuals genotyped.
(TIF)

S6 Fig LD-decay within *O. sativa*. Decay of linkage disequilibrium for all *O. sativa* samples, indica samples and japonica samples.
(TIF)

S7 Fig Average genome-wide diversity. Distribution of windows with different average number of pairwise differences within indica, within japonica, between indica and japonica, and global. The distribution within indica selective sweeps identified by [4] is also shown in yellow.
(TIF)

S8 Fig Comparison between NGSEP and mrCaNaVaR. A) Average number of basepairs in the Nipponbare reference with abnormal copy number variation predicted by NGSEP (blue), and mrCaNaVaR (red) for the indica and japonica populations. B). Percentage of the genome with abnormal copy number variation predicted by NGSEP also predicted by mrCaNaVaR.
(TIF)

S9 Fig Variability within a gene related to lethality. SNPs identified within the gene LOC_Os04g38950 in the 104 varieties analyzed in this study. Characteristic alleles of the temperate japonica haplotype are painted blue. Vertical dashed rectangles show the locations of the three missense SNPs with high minor allele frequency within indica. Colors in the left panel differentiate the following groups: *O. rufipogon* (RUF), aromatic (ARO), temperate japonica (TEJ), tropical japonica (TRJ), indica (IND), aus (AUS), *O. nivara* (NIV), and admixed (ADM).
(TIF)

S1 Table List of rice elite cultivars sequenced for this study. Database ids, names and relevance of the elite cultivars sequenced in this study. CIAT: International Center for Tropical Agriculture; LSU: Louisiana State University; YCGA: Yale Center for Genome Analysis; NCGR: National Center for Genome Resources; USDA-ARS: United States Department of Agriculture—Agricultural Research Service; IRRI: International Rice Research Institute. IRIS: International Rice Information System. GRIN: Germplasm Resources Information Network.
(XLS)

S2 Table Analysis of WGS data. Mapping statistics, specific pipeline parameters and structural analysis performed for each analyzed variety.
(XLS)

S3 Table Characteristic deletions within *O. sativa*. 100bp bins with characteristic deletions comparing indica, temperate japonica and tropical japonica.
(XLS)

S4 Table Characteristic duplications within *O. sativa*. 100bp bins with characteristic duplications comparing indica, temperate japonica and tropical japonica.
(XLS)

S5 Table Genome-wide individual population assignments. Map of population assignments for genomic stretches of 50 SNPs for the 104 varieties analyzed in this study.
(XLS)

S1 File Nexus dendogram file for 104 varieties. Nexus file including bootstrap values and branch lengths for the dendogram shown in Fig 1 including the 104 varieties analyzed in this study.
(NEX)

S2 File Nexus dendogram file for *O. sativa* varieties. Nexus file including bootstrap values and branch lengths for the dendogram shown in S3 Fig. A including the *O. sativa* varieties analyzed in this study.
(NEX)

S3 File Nexus dendogram file for indica varieties. Nexus file including bootstrap values and branch lengths for the dendogram shown in S3 Fig. B including the indica varieties analyzed in this study.
(NEX)

S4 File Nexus dendogram file for japonica varieties. Nexus file including bootstrap values and branch lengths for the dendogram shown in S3 Fig. C including the japonica varieties analyzed in this study.
(NEX)
