# Supplementary figures and images for: Whole Genome Sequencing of Elite Rice Cultivars as a Comprehensive Information Resource for Marker Assisted Selection
Source: PLoS One. 2015 Apr 29;10(4):e0124617. doi: 10.1371/journal.pone.0124617 (PMC4414565; doi:10.1371/journal.pone.0124617)

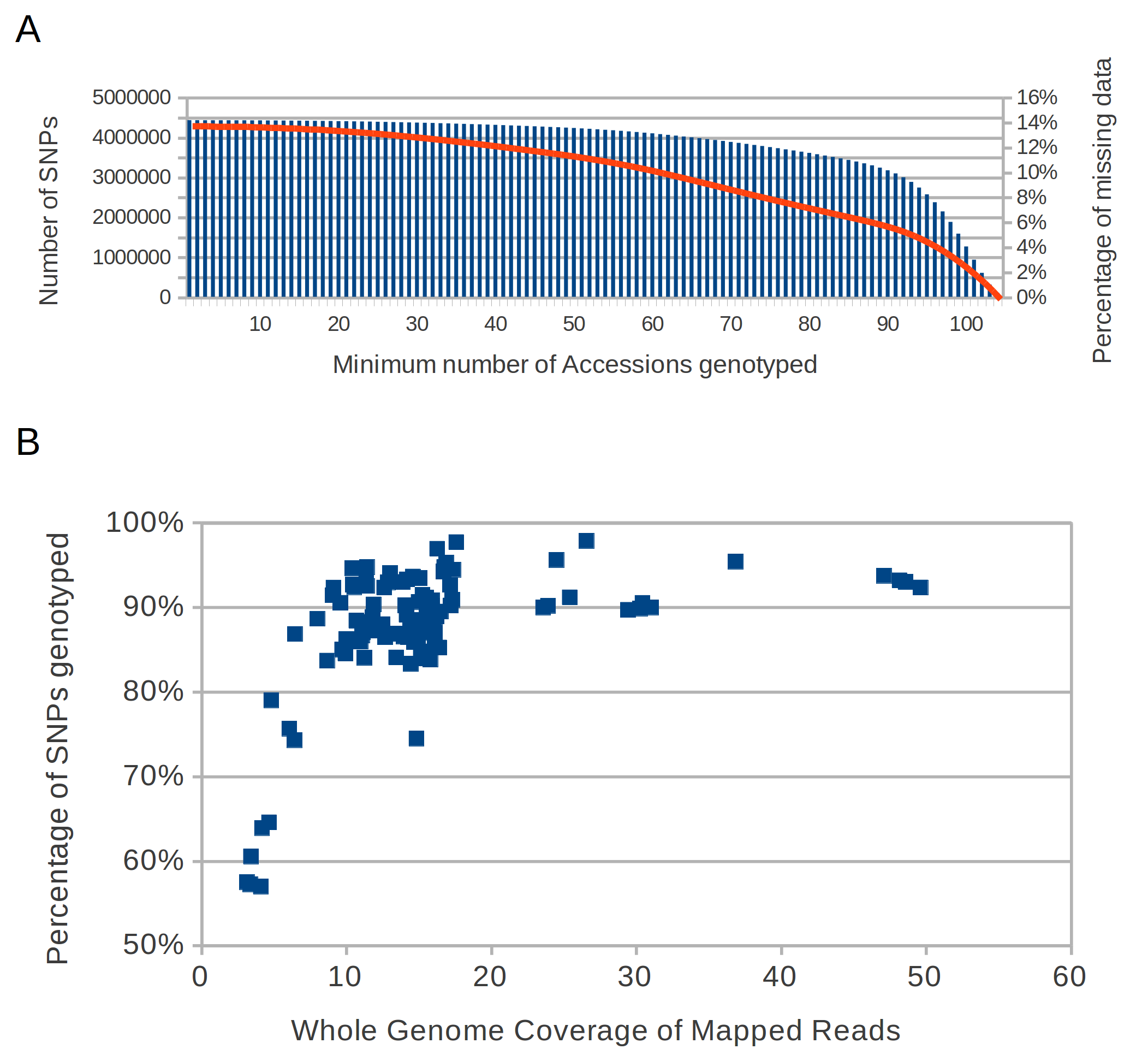

Supplement: S1 Fig — A) Number of SNPs obtained in non-repetitive regions (filter 1 in Table 1) for different minimum number of individuals genotyped. B) Percentage of SNPs genotyped as a function of the average coverage obtained from reads aligned to the Nipponbare reference genome. (TIF) [file pone.0124617.s001.tif]

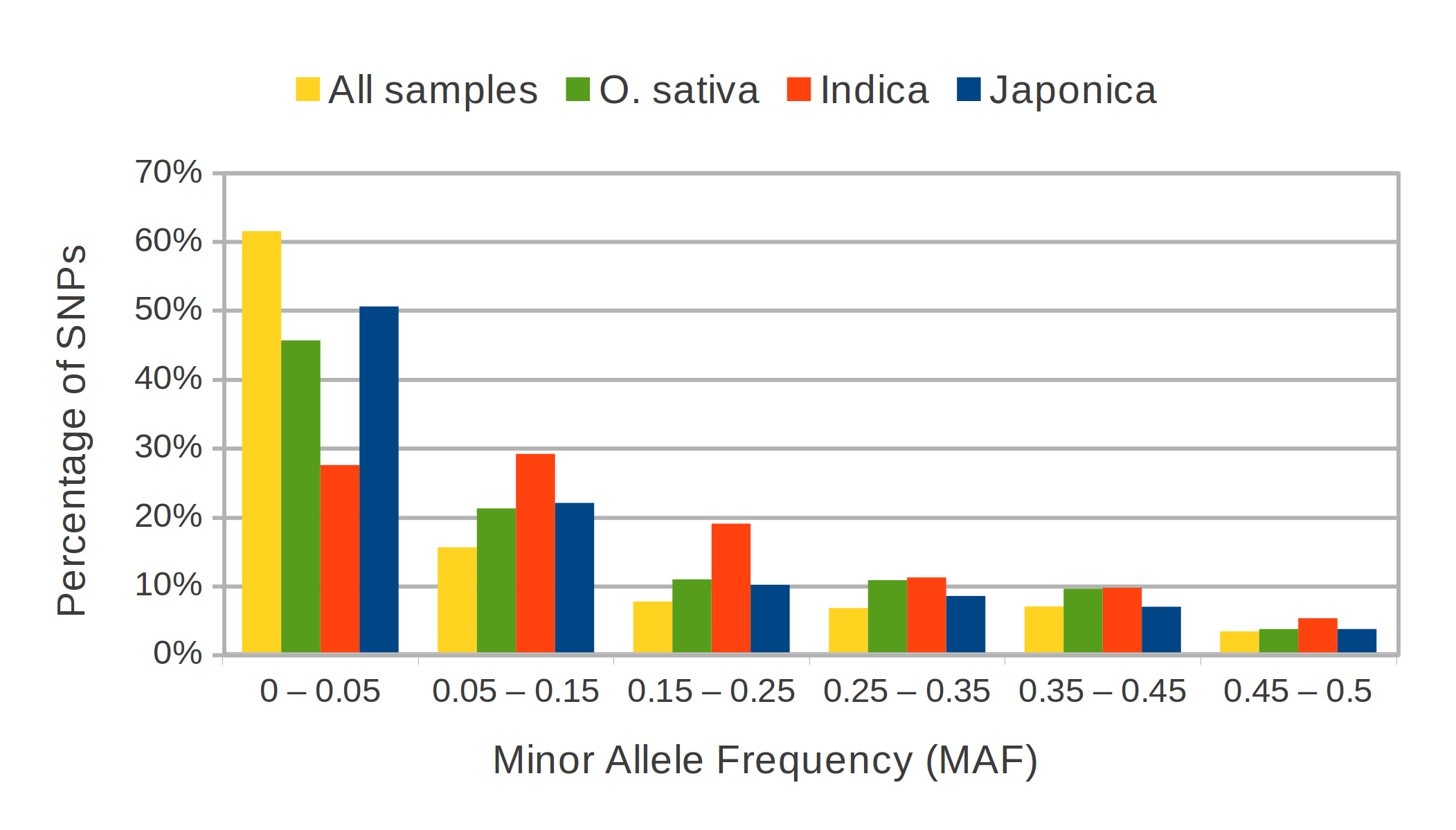

Supplement: S2 Fig — Distribution of allele frequencies for the SNPs found in non-repetitive regions (filter1 in Table 1) for the 104 varieties analyzed in this study (yellow bars) and for selected subsets based on membership to the O. sativa species (green), or membership to the two major subspecies within O. sativa, indica (red) and japonica (blue). (TIF) [file pone.0124617.s002.tif]

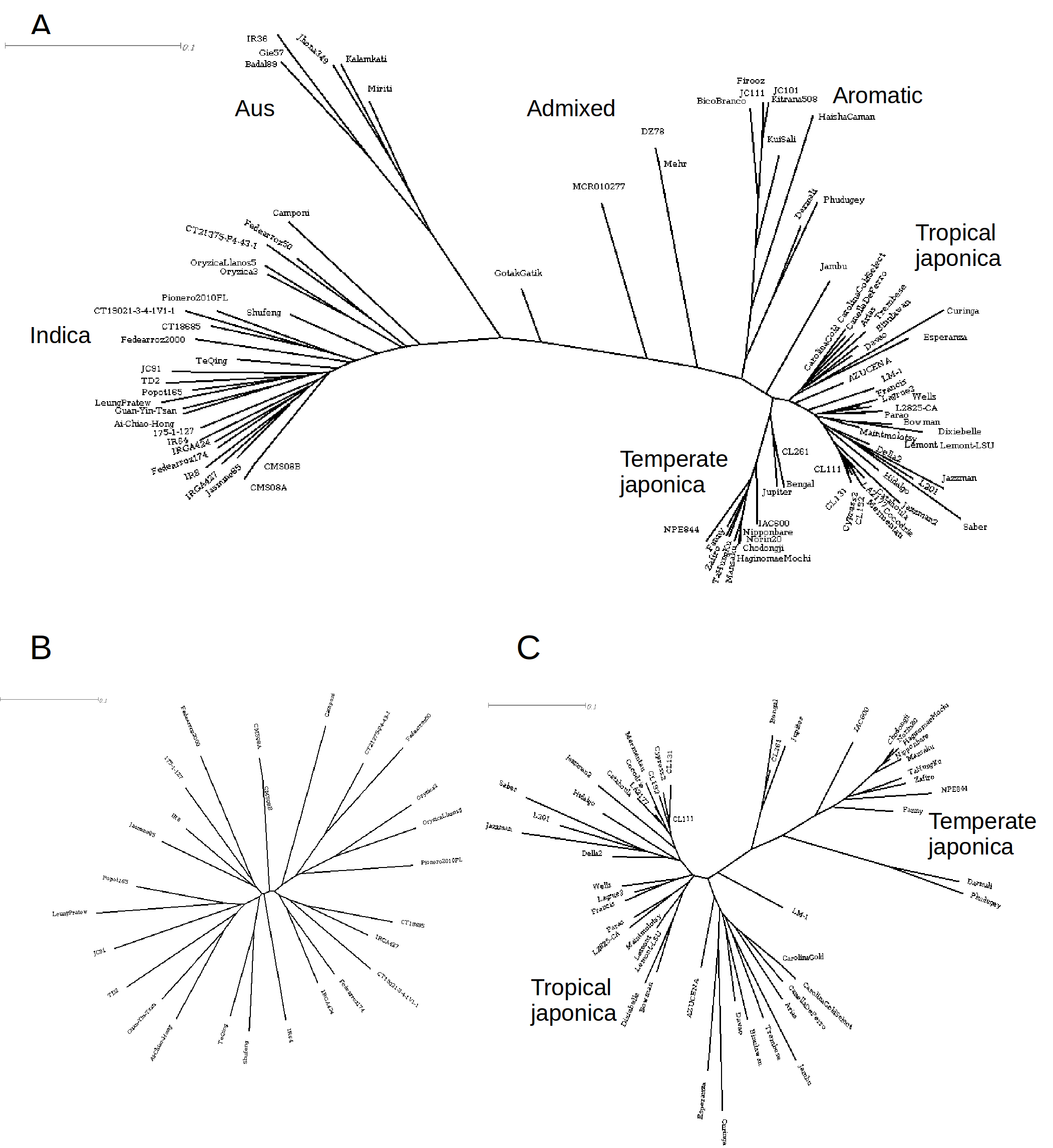

Supplement: S3 Fig — Neighbor joining dendograms for A) the 94 O. sativa varieties, B) indica varieties, and C) japonica varieties. (TIF) [file pone.0124617.s003.tif]

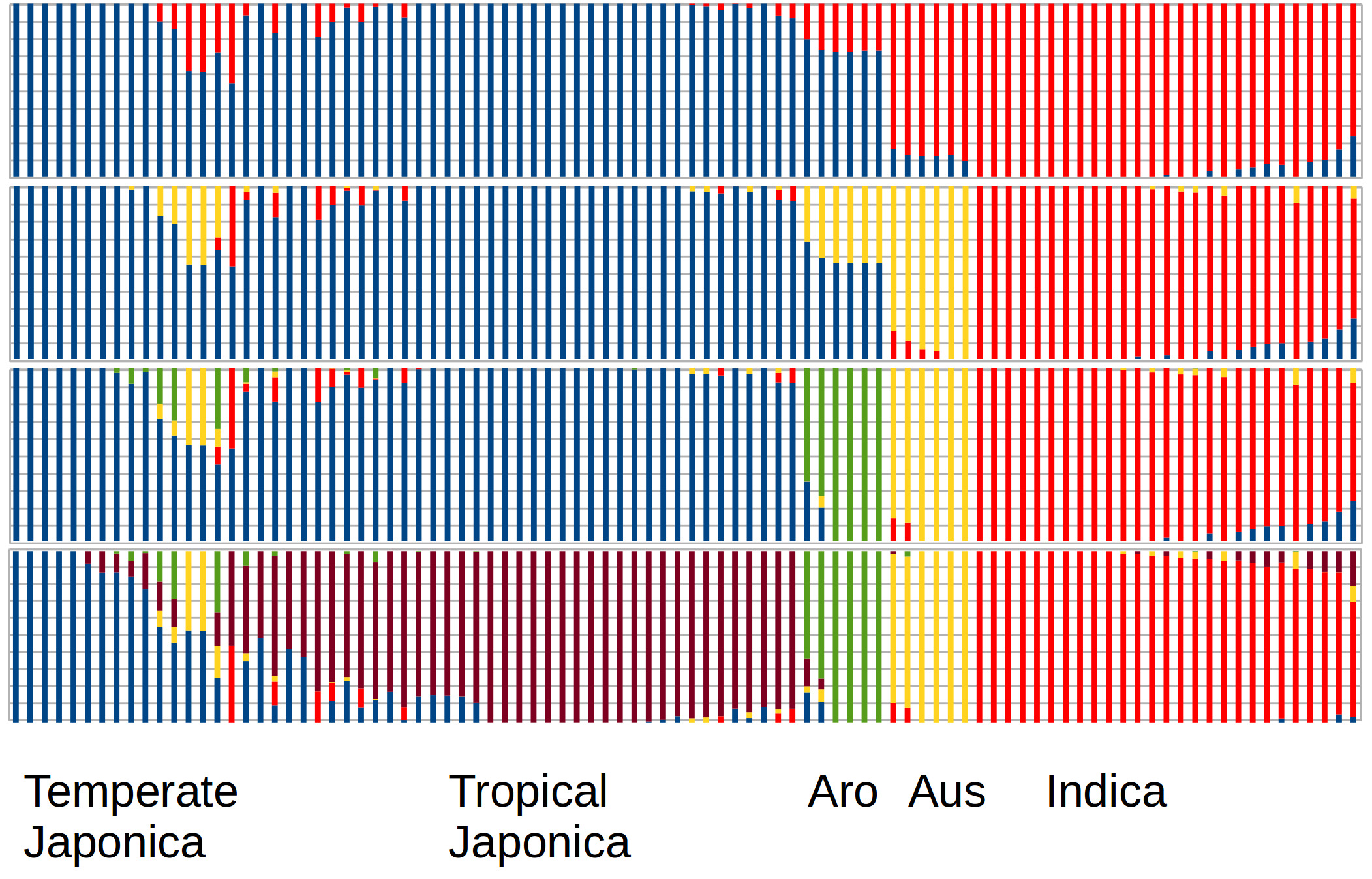

Supplement: S4 Fig — Clusters obtained with the Structure software changing the number of allowed populations (k parameter) from 2 to 5. (TIF) [file pone.0124617.s004.tif]

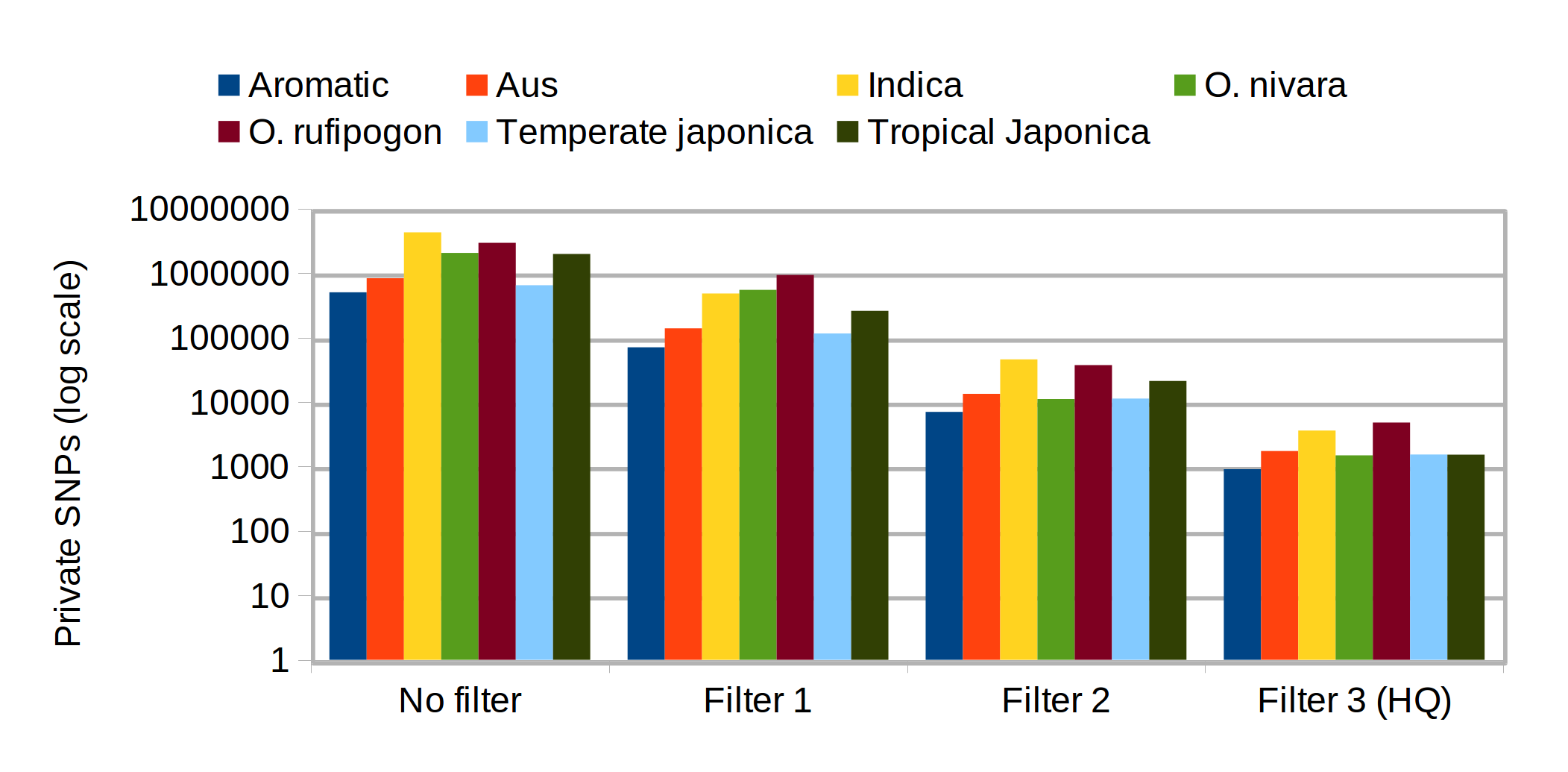

Supplement: S5 Fig — Number of SNPs polymorphic only within one population for the seven analyzed populations and for the following filtering strategies: 1) No filters 2) Remove SNPs within identified repetitive regions in Nipponbare, 3) Remove singleton SNPs (e.g. with the minor allele present in only one variety) and SNPs in regions in which at least three varieties report copy number variation, and 4) Remove SNPs in which at least one variety reports copy number variation, SNPs located less than 10 bp away from any other variant, and SNPs with less than 80 individuals genotyped. (TIF) [file pone.0124617.s005.tif]

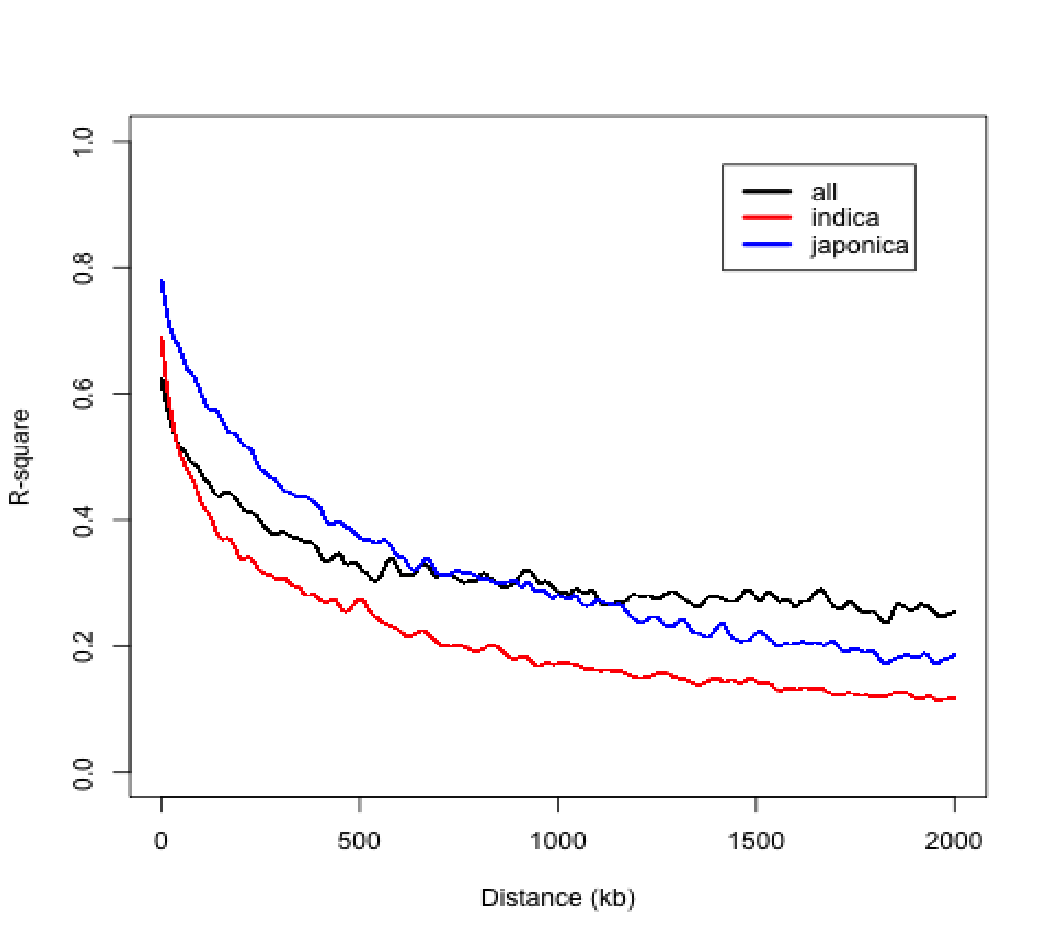

Supplement: S6 Fig — Decay of linkage disequilibrium for all O. sativa samples, indica samples and japonica samples. (TIF) [file pone.0124617.s006.tif]

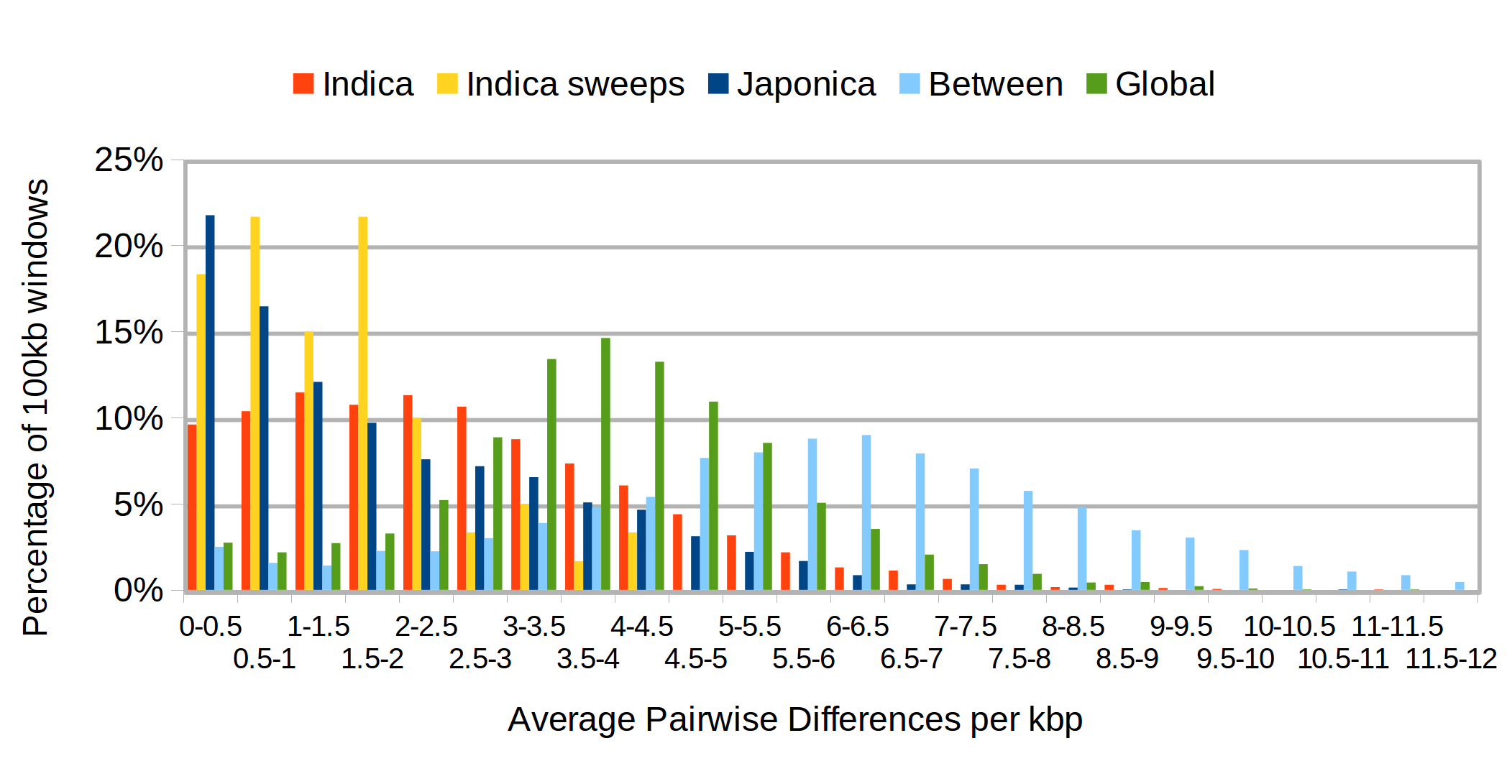

Supplement: S7 Fig — Distribution of windows with different average number of pairwise differences within indica, within japonica, between indica and japonica, and global. The distribution within indica selective sweeps identified by [4] is also shown in yellow. (TIF) [file pone.0124617.s007.tif]

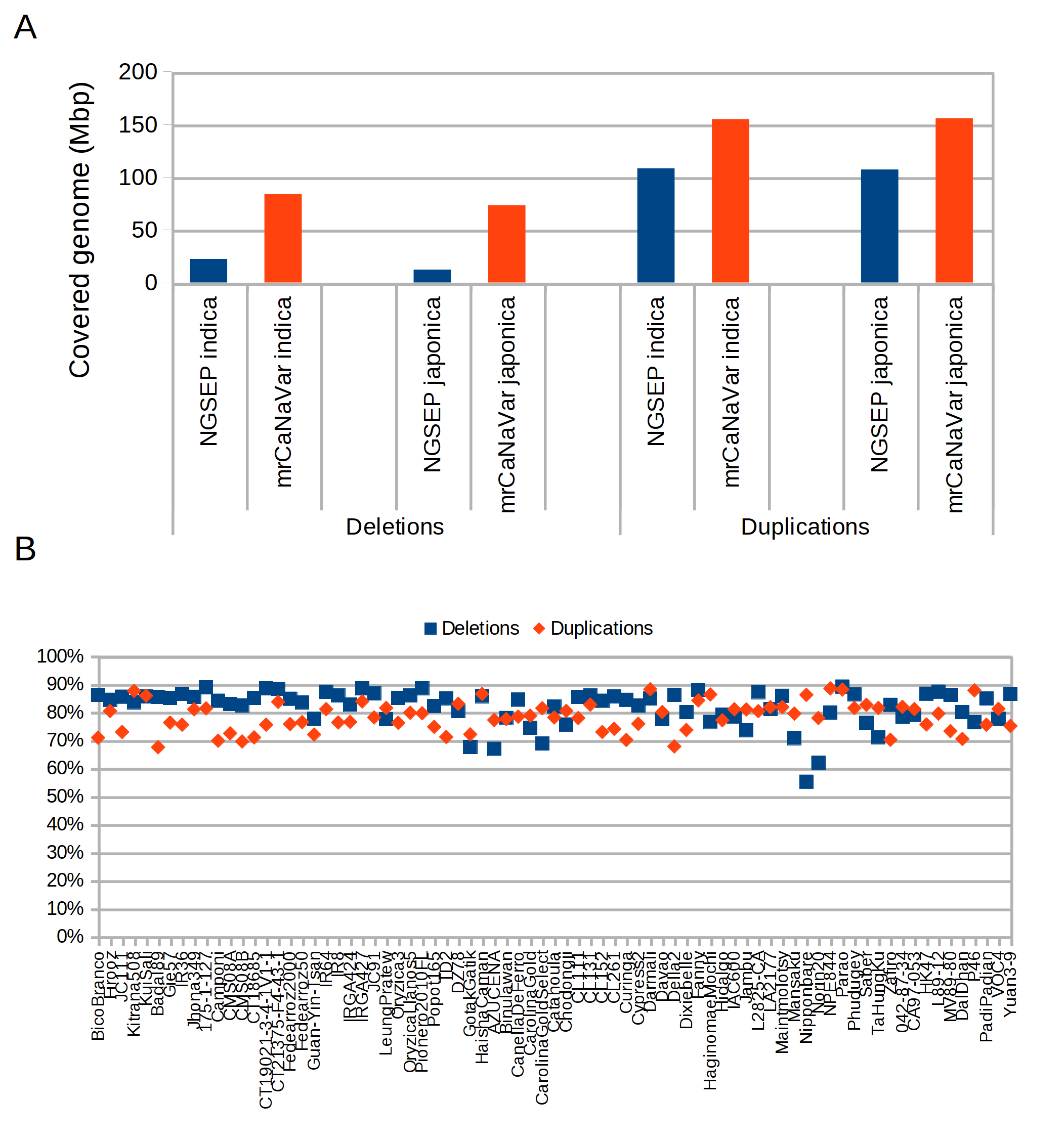

Supplement: S8 Fig — A) Average number of basepairs in the Nipponbare reference with abnormal copy number variation predicted by NGSEP (blue), and mrCaNaVaR (red) for the indica and japonica populations. B). Percentage of the genome with abnormal copy number variation predicted by NGSEP also predicted by mrCaNaVaR. (TIF) [file pone.0124617.s008.tif]

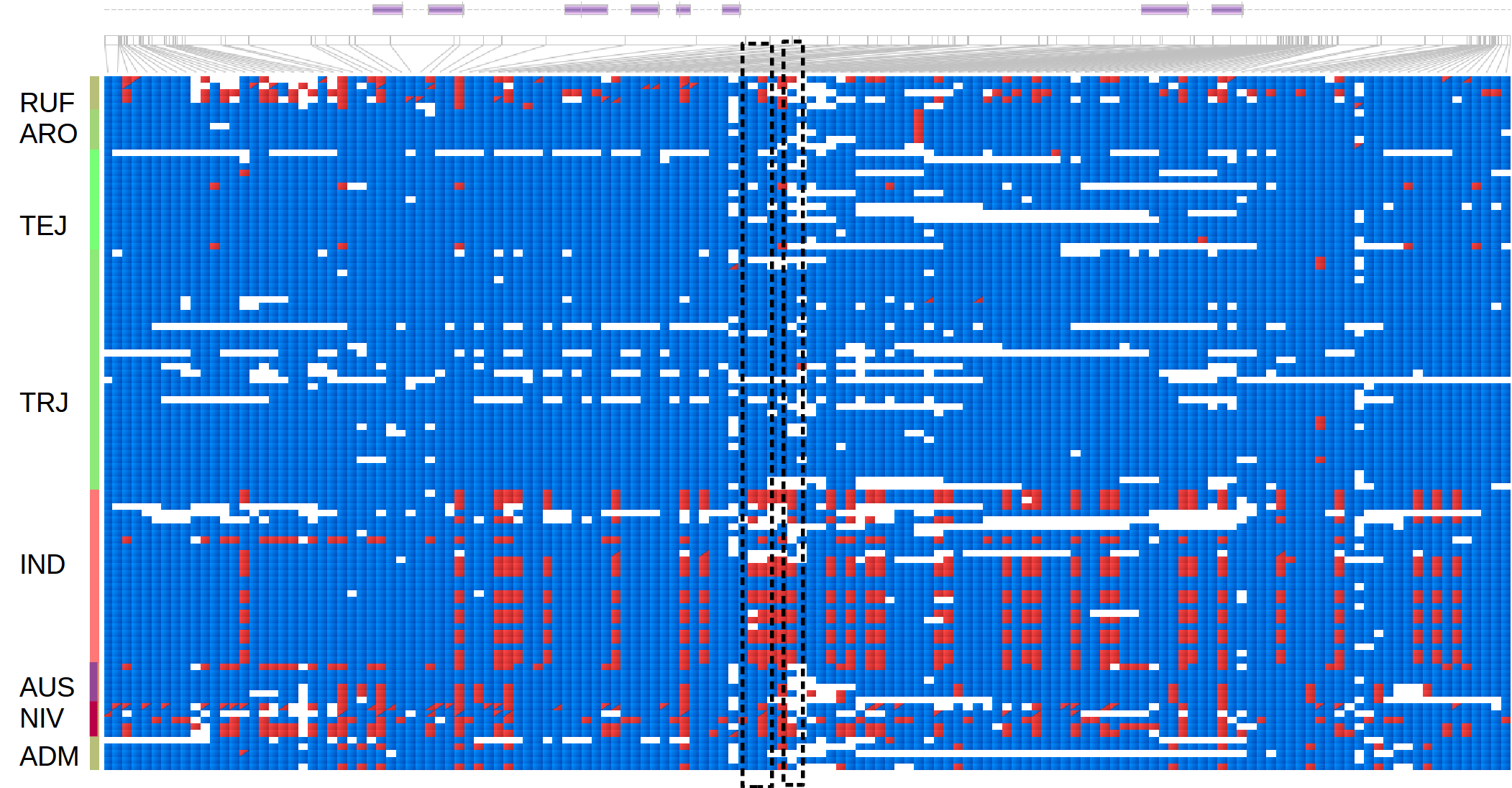

Supplement: S9 Fig — SNPs identified within the gene LOC_Os04g38950 in the 104 varieties analyzed in this study. Characteristic alleles of the temperate japonica haplotype are painted blue. Vertical dashed rectangles show the locations of the three missense SNPs with high minor allele frequency within indica. Colors in the left panel differentiate the following groups: O. rufipogon (RUF), aromatic (ARO), temperate japonica (TEJ), tropical japonica (TRJ), indica (IND), aus (AUS), O. nivara (NIV), and admixed (ADM). (TIF) [file pone.0124617.s009.tif]
